# Fallacies in Neuroscience: The Alzheimer's Edition

**DOI:** 10.1523/ENEURO.0530-21.2021

**Published:** 2022-02-10

**Authors:** Karl Herrup

**Affiliations:** Department of Neurobiology, School of Medicine, University of Pittsburgh, Pittsburgh, Pennsylvania 15213-2548

**Keywords:** Alzheimer’s disease, amyloid cascade hypothesis, β-amyloid, clinical trials, neurofibrillary tangles

In his editorial entitled “On Fallacies in Neuroscience,” Bernard (2020) takes his fellow neuroscientists to task for failures in logic that lead to the promulgation of fallacies and their entrenchment in the scientific literature. Drawing on his background in mathematics and philosophy, he highlights two common errors in logic that permeate our field.

The first error he discusses leads to a fallacy known as “reverse inference.” The example he cites is subtle, but all the more telling because it “feels” correct before we are shown the problem. Imagine that my brain is in a scanner when I experience a bout of fear. The scan shows that a particular brain region (Bernard calls it Area Z) lights up brightly with the onset of my fear. One would be correct in proposing that Area Z is likely involved in my brain's processing of fear. The fallacious conclusion, however, would be to assert that if Area Z is lit, I must be experiencing fear. That is the “reverse” of the experimental design, which was to induce fear and see which brain region activates. As Bernard (2020) points out, one could easily imagine that if I were experiencing a different emotion, even joy, Area Z might also light up. Without a lot more data, all we know is that the region is correlated with my experience of fear. We do not know that it is either necessary or sufficient to cause my fear.

The second error he describes leads to a fallacy known as “affirming the consequent.” His choice of example in this case is far easier to grasp. He starts with two objectively true statements. The first one is a conditional statement: if Francis Bacon wrote *Hamlet*, then he had to have been a great writer. The second is a simple, true observation: Francis Bacon was a great writer. Bernard then cautions us to avoid what is in this case an obvious logical fallacy: from these two facts alone, we cannot conclude that Francis Bacon wrote *Hamlet*.

As I read Bernard's short celebration of the importance of logical thinking, I was struck at how completely the field of Alzheimer's disease research has fallen into both fallacy traps he describes. The error of reverse inference can be seen in the very foundation of the amyloid cascade hypothesis. We know that people with Alzheimer's disease have plaques and tangles in their brains. Plaques and tangles are associated with Alzheimer’s disease. However, just as we cannot conclude that Area Z causes fear, we are not entitled to conclude that plaques and tangles cause Alzheimer’s disease. It is OK to use these data to form and state a hypothesis: plaques and tangles might cause Alzheimer’s disease. Indeed, that is precisely what Hardy and Higgins (1992) did in their first exposition of the amyloid cascade hypothesis. The trap that the field has fallen into is to use reverse inference to turn this hypothesis into dogma.

The second fallacy is equally prevalent in the field. Rather than Bacon wrote *Hamlet*, we instead have very wise people telling us that amyloid and tau cause Alzheimer’s disease. This is supported, largely, by the same logic that was used above to deny Shakespeare credit for writing *Hamlet*. We start with two objectively true statements. First, the conditional: if plaques and tangles cause Alzheimer’s disease, then their constituents [aggregated β-amyloid (Aβ) and/or tau] must be neurotoxic. The second is a short, objectively true observation: Aβ and tau are neurotoxic. These two facts are consistent with the hypothesis that plaques and tangles cause Alzheimer’s disease, but they are not proof.

As I detail in my new book (Herrup, 2021), we have acquired a great deal of data since the first exposition of the idea of an amyloid cascade, and most of it tends to falsify the original hypothesis. Yet, it persists in the literature, indeed dominates it, not as a hypothesis but as dogma. As I have argued before (Herrup, 2015), the field has tested two of its most basic predictions and they have failed. We have added tau and amyloid to the brains of healthy mice and humans, yet the predicted onslaught of Alzheimer’s disease either does not occur or is so slow to appear that common sense would have us search for other, perhaps more direct causes. We have also removed tau and amyloid from the brains of people with Alzheimer’s disease, yet they continue their cognitive decline (Mullard, 2021a, b; Reiss et al., 2021). We still debate whether the decline is less steep than in untreated persons, but under even the most optimistic readings of the data, the effect of removing amyloid or tau is modest.

To these two fallacies, I would add a third: the assumption of causality from an observed temporal sequence. As I do not have a background in philosophy, I do not know whether there is a formal name for this error. The fallacy is best exemplified by the famous diagram shown in Figure 1. Listing the S-shaped curves from left to right provides the following sequence: Aβ in CSF precedes PET-detectable amyloid deposits, which precede tau in CSF, which precedes reductions in brain activity or volume, which precede cognitive impairment. This list is true (although the shapes of the curves are somewhat speculative). But just because Aβ comes before tau in time does not mean that Aβ causes the changes in tau. That can be seen simply by my modified version of the figure (Fig. 2). I am being ridiculous to make the logical fallacy clear. My revised sequence of events is objectively true. Yet no one would argue that gray hair causes the rise in CSF Aβ or any of the other changes in the diagram.

**Figure 1.**
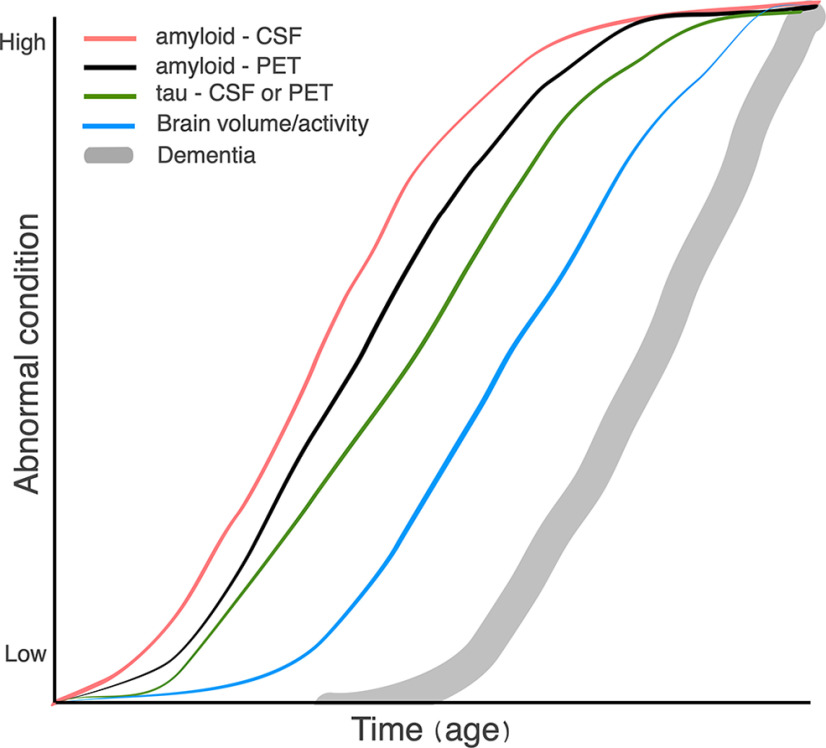
Average rates of appearance of amyloid and tau markers in Alzheimer's disease (after Jack et al., 2013).

**Figure 2.**
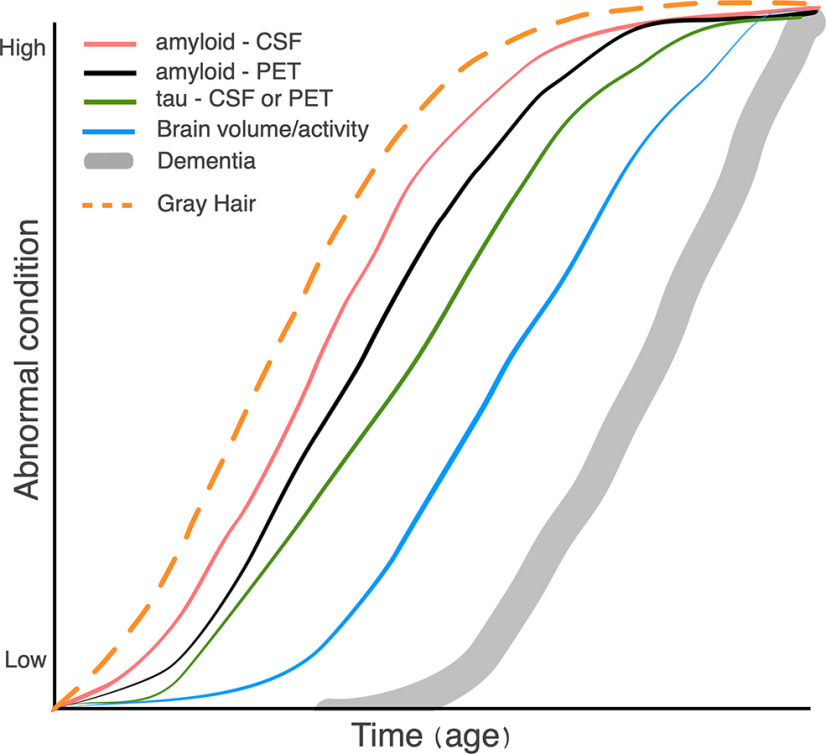
Modification of Figure 1.

Stated as a hypothesis of the importance of amyloid in the pathogenesis of Alzheimer’s disease, Figure 1 is as useful as the original statement of the amyloid cascade hypothesis. But it is only useful in a seminar or publication if it is clearly identified as a hypothesis. To treat it as fact is the same error as arranging a line of 20 random stones in order of increasing size, then using a photograph of the arrangement to prove that stones grow.

The question before us then is what can be done to reduce the burden that these fallacies place on the field; what is our path forward? I must admit that I am rather optimistic about our prospects for finding new Alzheimer’s disease treatments. Despite a string of new publications arguing for the continued value of the amyloid cascade hypothesis (for review, see Selkoe, 2021), novel and exciting nonamyloid alternatives are increasingly being developed and tested, even as anti-tau and anti-amyloid treatments remain a significant part of our clinical trial portfolio. That said, I cannot shake a deep pessimism from what I perceive as the near futility of hoping that our field will ever honestly address the logical weaknesses of applying the amyloid cascade hypothesis to the pathogenesis of Alzheimer’s disease. I am reminded of the old adage that holds that it is hard to make a person understand something if their salary depends on their not understanding it. For the pharmaceutical industry, the “salary” is the deep financial investments that they have made in therapeutic approaches based on the amyloid cascade. For the National Institutes of Health and many of the private charities that fund Alzheimer’s disease research, the salary is not only their financial commitments, but also their international reputation. These groups have repeatedly told investors, politicians, and donors that they know how to get this done. If amyloid is not the cause of Alzheimer’s disease, there will be a lot of very uncomfortable explaining to be done.

There are arguments that can be made for continuing to pursue anti-amyloid and anti-tau approaches: “we just need to start earlier in the disease process,” or “we just need to pick our trial population more carefully,” or “we just need to design our trials more efficiently,” etc. What is clear, however, is that our field lacks the courage to answer one simple question: what experimental result could we agree on that would falsify the amyloid cascade hypothesis? If the answer to that question is “none,” then it is not a hypothesis and has no logical value. In thus abandoning logic and clinging to dogma, my fear is that Alzheimer’s disease research will be mired in fallacies for some time to come. And the costs—in lost time, lost money, and lost lives—will be enormous.

## Synthesis

Reviewing Editor: Christophe Bernard, INSERM & Institut de Neurosciences des Systèmes Decisions are customarily a result of the Reviewing Editor and the peer reviewers coming together and discussing their recommendations until a consensus is reached. When revisions are invited, a fact-based synthesis statement explaining their decision and outlining what is needed to prepare a revision will be listed below. The following reviewer(s) agreed to reveal their identity: NONE.

N/A

## References

[B1] Bernard C (2020) On fallacies in neuroscience. eNeuro 7:ENEURO.0491-20.2020. 10.1523/ENEURO.0491-20.2020 PMC7729298 33303561

[B2] Hardy JA, Higgins GA (1992) Alzheimer’s disease: the amyloid cascade hypothesis. Science 256:184–185. 10.1126/science.1566067 1566067

[B3] Herrup K (2015) The case for rejecting the amyloid cascade hypothesis. Nat Neurosci 18:794–799. 10.1038/nn.4017 26007212

[B4] Herrup K (2021) How not to study a disease: the story of Alzheimer’s. Cambridge, MA: MIT.

[B5] Jack CR, Knopman DS, Jagust WJ, Petersen RC, Weiner WW, Aisen PS, Shaw L, Prashanthi V, Wiste HJ, Weigand SD, Lesnick TG, Pankratz VS, Donohue MC, Trojanowski JQ (2013) Tracking pathophysiological processes in Alzheimer’s disease: an updated hypothetical model of dynamic biomarkers. Lancet Neurol 12:207–216. 10.1016/S1474-4422(12)70291-0 23332364 PMC3622225

[B6] Mullard A (2021a) Failure of first anti-tau antibody in Alzheimer disease highlights risks of history repeating. Nat Rev Drug Discov 20:3–5. 10.1038/d41573-020-00217-7 33303932

[B7] Mullard A (2021b) Anti-tau antibody failures stack up. Nat Rev Drug Discov 20:888. 10.1038/d41573-021-00187-4 34728770

[B8] Reiss AB, Montufar N, DeLeon J, Pinkhasov A, Gomolin IH, Glass AD, Arain HA, Stecker MM (2021) Alzheimer disease clinical trials targeting amyloid: lessons learned from success in mice and failure in humans. Neurologist 26:52–61. 10.1097/NRL.0000000000000320 33646990

[B9] Selkoe DJ (2021) Treatments for Alzheimer’s disease emerge. Science 373:624–626. 10.1126/science.abi6401 34353940

